# Clinical Approach to Acute Decline in Sensorium

**DOI:** 10.5005/jp-journals-10071-23188

**Published:** 2019-06

**Authors:** Vasudha Singhal

**Affiliations:** Department of Neuroanesthesiology and Critical Care, Medanta – The Medicity, Gurugram, Haryana, India

## Abstract

**How to cite this article:** Singhal V. Clinical Approach to Acute Decline in Sensorium. Indian J Crit Care Med 2019;23(Suppl 2):S120–S123.

## INTRODUCTION

Acute decline in sensorium is a commonly encountered symptom in the neurocritical care units, the differential for which is enormous. The causes may range from easily reversible (such as hypoglycemia) to relatively permanent (such as stroke), and from benign (such as intoxication) to potentially life threatening (such as meningo-encephalitis) etiologies. A streamlined approach to such patients is necessary for a systematic diagnostic workup and appropriate management.

## PATHOPHYSIOLOGY

A decrease in the level of consciousness of a patient may typically result either from a disruption of the *ascending reticular activating system*, a neuronal network running from the brainstem to thalamus to the cerebal cortex, controlling arousal or an impairment in the bilateral cortices, where the sensory processing occurs, generating awareness.^1^

The fall in sensorium is due to a diffuse neuronal dysfunction caused by a decreased supply of glucose and oxygen to the brain, from either structural or non-structural brain diseases. Structural causes of a decline in sensorium include those that cause focal pressure in the brain, ultimately blocking substrate delivery at the cellular level. They include – trauma (subdural or epidural hematoma), brain tumors, intracranial hemorrhage, hydrocephalus, vascular occlusion etc. Patients with a decline in sensorium due to a structural cause usually have asymmetrical neurological findings, such as anisocoria, hemiparesis, asymmetric eye movements etc. An urgent imaging (computed tomography, CT head) is required to exclude a potential herniation syndrome or stroke, that need urgent intervention.^2^

Non-structural causes result in substrate disruption at the cellular level due to toxic and metabolic etiologies. Exogenous toxins, or an endogenous perturbation of the metabolic milieu (such as sodium imbalance or dysglycemia), may result in a decline in sensorium, with symmetric or generalized examination findings. However, lesions involving the brainstem or the diencephalic arousal centres may also result in symmetric findings.

The common etiologies causing an acute decline in sensorium can be classified into neurological causes (which may be structural or non-structural), or toxic metabolic causes (non-structural).^3^

### Neurologic Causes

- Trauma – epidural/ subdural/ intracranial hematomas*, diffuse axonal injury
- Tumors* – primary central nervous system (CNS)/ metastatic
- Vascular – strokes (ischemic/ hemorrhagic/ subarachnoid hemorrhage)*, hypoxic ischemic encephalopathy
- Infections – meningitis/ encephalitis, brain abscess*
- Seizures – postictal/ nonconvulsive status epilepticus
- Acute hydrocephalus due to any cause
- Others–
  – Posterior reversible encephalopathy syndrome (PRES)
  – Autoimmune encephalitis
  – Osmotic demyelination syndrome

‘*’ indicates ‘primarily structural causes resulting in asymmetrical neurological findings’

### Toxic-metabolic Causes

- Toxic – drug overuse
  – Narcotics
  – Sedative-hypnotics
  – Drugs of abuse – alcohol, opioids, amphetamine, cocaine
  – Medication overdose – tricyclic antidepressants, selective serotonin reuptake inhibitors (SSRI), anticonvulsants, antipsychotics, acetaminophen, aspirin
- Metabolic encephalopathy –
  – Hypoxia / hypercapnia
  – Dysglycemia – hypoglycemia or hyperglycemia (diabetic ketoacidosis/ hyperosmolar non-ketotic hyperglycemia)
  – Sepsis
  – Shock / hypoperfusion states
  – Hypo / hypernatremia
  – Hepatic encephalopathy
  – Uremia
  – Wernicke's encephalopathy
  – Endocrine etiology – myxedema / adrenal insufficiency/ hypercalcemia
- Environmental causes – carbon monoxide poisoning / heat stroke/hypothermia

## INITIAL ASSESSMENT

The initial approach to a patient with an acute alteration in mental status should focus on stabilizing the patient. A quick **‘ABCDE approach’** not only helps in patient stabilization, but also aids in excluding many reversible causes of decreased sensorium.

Managing **A, airway,** and **B, breathing,** help in correcting hypoxia, causing a decline in sensorium. Decision regarding airway management with endotracheal intubation is however, ambiguous, keeping in mind the quick reversibility of certain causes of altered sensorium, such as hypoglycemia. While a Glasgow Coma Scale (GCS) <8 is considered an indication for intubation, some patients who remain in an acute care area may be managed expectantly, such as patients with alprazolam overdose. On the other hand, a patient with a structural lesion, such as an intracranial hemorrhage, showing an acute decline in sensorium from a GCS of 14 to 8, may need urgent intubation and mechanical ventilation. Concurrent with airway management, care should be taken to immobilize the cervical spine, if there is a suspicion of injury.

**C, circulation,** is important to rectify hypotension and look for arrhythmias. Presence of hypertension may point towards the possibility of a severe intracranial hypertension and an impending herniation, and should be treated immediately. Cardiac monitoring may be needed in patients who presented with arrhythmias causing hypoperfusion and an acute decline in sensorium (syncope in most cases).

Assessing **neurological disability ‘D’** is one of the most important steps in the evaluation of patients with altered sensorium. It includes – assessment of the level of consciousness using the GCS or the alert verbal painful unresponsive (AVPU) score, pupillary size and reaction (to rule out space occupying lesions in the brain), motor power in the limbs (hemiparesis in stroke), involuntary movements (seizures), and brainstem reflexes.

**E or ‘expose’** is to perform a quick head to toe examination to look for signs of trauma, petechiae, infectious sources such as indwelling catheters, needle pricks in intravenous drug abusers, or transdermal drug patches.

### What Next?

A quick intravenous access is established while managing the ABC, and blood sent for investigations simultaneously (serum chemistries, basic hematologic panel, arterial blood gas, ammonia, toxicology screens). Bedside blood glucose is performed in all patients to exclude hypoglycemia, a life threatening but quickly reversible cause of an altered mental status. If the blood sugar is <70 mg/dL, 50 mL of 50% dextrose intravenous (iv) is administered, along with thiamine 100 mg iv to prevent the precipitation of acute Wernicke's encephalopathy (especially in patients with a risk of nutritional deficiency, such as chronic alcoholics and malnourished patients).

A good history taking is important to know the patient's concurrent medical illnesses (such as diabetes, hypertension, renal failure, seizures, hypothyroidism, psychiatric issues), any history of trauma, fever or headaches, possible toxic ingestion, medication history, etc. A focussed physical examination should proceed simultaneously and vital signs should be checked, including pulse oximetry, heart rate and rhythm, blood pressure, respiratory rate and temperature. The vital signs may give significant leads to the cause of the altered sensorium–

- *Heart rate*: Bradycardia may be seen in cases with raised intracranial pressure (ICP), and in medication overdose with sympatholytic drugs (eg clonidine) or sedative hypnotics (e.g. barbiturates). Tachycardia is common in patients with sepsis (presenting with hypotension), intracranial hemorrhage (due to sympathetic overactivity), psychotropic drug poisoning, and ketamine overdose.
- *Blood pressure*: Hypotension is a common presenting feature of patients with sepsis. Drug overdosage (e.g. tricyclic antidepressants, sedative-hypnotics, clonidine) may also present with hypotension. Hypertension, on the other hand, may be seen as a part of the Cushing's reflex in patients with raised ICP, or in patients presenting with hypertensive encephalopathy (PRES). It is also common in ketamine and phencyclidine intoxication.
- *Respiratory rate*: Bradypnoea is seen in opioid and sedative-hypnotic overdose. Bradypnoea in the setting of a structural neurological disease, points towards a medullary involvement, and is an ominous sign. Tachypnoea is common in patients presenting with metabolic acidosis due to any cause – diabetic ketoacidosis, uremia, alcoholics, drug overdose, sepsis and so on.
- *Temperature*: Hyperthermia may be reflective of a septic etiology for the altered sensorium. Fever may also be seen in patients with subarachnoid hemorrhage (SAH), hypothalamic injury, salicylate poisoning, or environmental exposure. Hypothermia is usually due to an environmental cause. Other causes include severe sepsis, hypoglycemia, hypothyroidism, or sedative-hypnotic overdose.

If, at any stage, there is a clinical suspicion of opioid toxicity (history of illicit drug use, apnoea or bradypnoea, pinpoint pupils), iv naloxone 0.4–2 mg should be administered and repeated upto 4 mg if necessary.

In patients presenting with clinical signs and symptoms of sepsis, blood cultures should be drawn and broad spectrum antibiotics initiated.

All this while, one must also keep in mind functional etiology of an acute decline in sensorium. Presence of a firm resistance to eye opening is a reasonably sensitive and specific sign of functional unresponsiveness. The eye gaze sign, in which the patient tends to look to the ground when turned on one side, may also act as a clue.^4^ Also, when the patient's arm suspended over his/her face is released, it tends to move away and doesn't fall over the face.

## DETAILED NEUROLOGICAL ASSESSMENT

## Level of Consciousness

The level of consciousness is most commonly assessed by the GCS across all intensive care units.^5^ The GCS is simple, reproducible and reliable in documenting a patient's response (eye opening, verbal and motor response). But it does not account for alterations in brainstem function, hemiparesis or aphasia, and is not reliable in intubated patients. A simplification of the GCS to measure the level of consciousness, mostly in emergency medicine protocols, is the AVPU score (Alert, Verbal or voice responsive, Pain responsive, Unresponsive).^6^ This however, cannot be used for long term follow-up of neurological status. The Full Outline of UnResponsiveness (FOUR) score is a new scoring system that incorporates brainstem function and respiratory pattern (instead of the verbal score), and can be used reliably even in the intubated patients in ICU.^7^ The FOUR score assesses the neurological status under 4 domains: eye opening, motor response, brainstem reflexes (pupillary, corneal and cough reflexes), and respiratory pattern. Each domain has a score of 0–4, making a total maximum of 16. The FOUR score is considered a better predictor of mortality in critically ill patients.^8^

### Brainstem Reflexes

Careful assessment of brainstem reflexes such as the pupillary reflex (size, reaction to light, symmetry), corneal reflex, gag and cough reflexes provide information about the functioning of the various components of brainstem. Other reflexes include the oculo-cephalic reflex, vestibulo-ocular reflex, visual threat response etc.

Dilated, non-reactive pupils are indicative of third nerve palsy due to midbrain compression, seen in lesions causing intracranial hypertension (head trauma, hemorrhage, cerebral edema, hydrocephalus etc). Drug intoxication with tricyclic antidepressants may also cause mydriasis. Constricted or pin-point pupils are seen in pontine lesions (infarct/hemorrhage) and in opiate poisoning. Horizontal nystagmus may be seen in alcoholics and antiepileptic drug intoxication. Vertical nystagmus may suggest brainstem dysfunction. Gaze deviation is seen with ipsilateral hemispheric lesions, contralateral pontine lesions, or a seizure focus. Fundoscopy may reveal retinal hemorrhages (seen in trauma and hypertension) or papilloedema (in raised ICP). Brainstem assessment may also play an important role in diagnosing posterior circulation strokes such as basilar occlusion syndromes, which are an important differential in a patient with an acute decline in sensorium.^9^

### Motor Response

The patients are assessed for spontaneous limb movements, hemi- or para-paresis/plegia, and posturing. Symmetric posturing may be either decorticate or decerebrate, and point towards an involvement of the brainstem or diencephalic arousal centres. Motor function is assessed in response to verbal commands, or painful stimulation. Muscle tone of the extremities is evaluated by passive limb movement. Deep tendon and cutaneous reflexes are checked for briskness and symmetry.

### Breathing Patterns

Respiratory patterns may be useful in localizing the source of altered sensorium. Central neurogenic hyperventilation is seen in lesions of the pons / midbrain. Apneustic or ataxic breathing are suggestive of medullary involvement and are an indication for endotracheal intubation. Cheyne stokes respiration is seen in patients with raised intracranial pressure (including trauma, stroke, brain tumor etc.), toxic metabolic encephalopathy, and carbon monoxide poisoning.

Following this initial work-up, patients with a decline in sensorium are triaged into structural and non-structural arms, so as to facilitate early involvement of the surgical team in cases of structural lesions for either decompression or invasive neuromonitoring.

## IMAGING AND LABORATORY TESTING

Unless a recognizable infectious or metabolic cause for altered mentation is established, additional testing including imaging is necessary. A CT head helps in ruling out a structural lesion of the brain, particularly in the setting of trauma. Magnetic resonance imaging (MRI) brain with contrast may yield greater definition of cortical and subcortical structures, and may show white matter lesions, acute infarcts, peri-tumor edema, leptomeningeal enhancement etc. Advanced vascular imaging such as CT angiography or MR angiography may be indicated in patients suspected to have an ischemic stroke (basilar artery strokes cause sudden unresponsiveness).

The basic laboratory investigations in patients with a decline in sensorium include, complete blood count (leucocytosis in sepsis), serum glucose (to rule out hypo/ hyperglycemia), ECG (to rule out arrhythmias causing syncope, or conduction abnormalities in dyselectrolytemias), serum electrolytes including calcium, renal and hepatic function tests, and serum ammonia (to rule out hepatic encephalopathy). An arterial blood gas analysis may be helpful in toxic causes of altered sensorium.

Electroencephalography (EEG) is an important diagnostic tool in excluding non-convulsive status epilepticus as a cause of fall in sensorium. It is indicated in patients with a history of seizures.

Lumbar puncture may be indicated in suspected cases of meningo-encephalitis. A history of fever, stiff neck or photophobia may be suggestive of a CNS infection.

## TREATMENT

The treatment of a decline in sensorium is guided by its underlying etiology. However, the first priority in all patients presenting with an altered sensorium, irrespective of etiology, is to provide general supportive care. In fact, this may be the only therapeutic modality that can be offered to the patient, e.g. in cases of hypoxic ischemic encephalopathy, or in toxin induced diffuse neuronal dysfunction. Securing airway with intubation, mechanical ventilation to ensure adequate oxygenation, and use of intravenous fluids and vasopressors for resuscitation form the basis of the general supportive care. Wherever appropriate, specific antidotes or antagonists are administered e.g. naloxone in opioid intoxication, N-acetyl cysteine in acetaminophen poisoning etc. Flumazenil (0.2 mg iv, repeated to a maximum of 1 mg) may be considered to reverse respiratory depression in patients with severe, isolated benzodiazepine toxicity, especially in accidental pediatric ingestions or reversal of iatrogenic oversedation.^10^

Fall in sensorium due to structural neurological causes may be life threatening, and need urgent neurosurgical intervention to improve long term outcomes. Urgent NCCT (non-contrast CT) head guides surgical decompression and/or ventriculostomy, if need be. Patients with ischemic stroke (posterior circulation strokes usually cause an acute decline in sensorium) are offered intravenous thrombolysis ± endovascular thrombectomy depending upon eligibililty.

In patients with metabolic encephalopathy, the treatment target is to achieve homeostasis. In some cases e.g. hypoglycemia, a rapid normalization of levels (in this case, blood glucose) reverses symptoms. In other cases, only partial correction of the parameter is indicated to prevent the precipitation of adverse events. E.g. a rapid increase of sodium levels in acute symptomatic hyponatremia may lead to osmotic demyelination; aggressive lowering of blood pressure to normal levels in hypertensive encephalopathy may result in acute stroke due to a decreased cerebral perfusion.

In patients with toxin induced encephalopathy, alteration of systemic or compartmental pH may be indicated to reduce drug toxicity or increase drug excretion. For example, in aspirin poisoning, making the urine more alkaline with sodium bicarbonate increases ionisation of the salicylic acid, thereby increasing its excretion from the body. Also, intravenous lipid emulsions have been used to alleviate toxicity of many lipophilic drugs like cocaine, methamphetamine etc, and help in restoring hemodynamic and neurological stability in affected patients by altering drug distribution.^11^

## SUMMARY

The causes for an acute decline in sensorium may be numerous, ranging from drug intoxication, to metabolic encephalopathy, to a wide variety of neurological diseases. The pathology is either in the ascending reticular activating system, or is bilateral and diffuse in the hemispheres. Patients presenting to neurocritical care units usually have structural causes, like traumatic brain injury, intracranial hemorrhage, brain tumors, hydrocephalus etc, or a metabolic cause such as hyponatremia. Neurological worsening may also result from sepsis, meningitis and seizures. The general approach is initial stabilization of the patient, followed by treatment of the underlying etiology.

## References

[1] Edlow BL,, Takahashi E,, Wu O. (2012;). Neuroanatomic connectivity of the human ascending arousal system critical to consciousness and its disorders.. J Neuropathol Exp Neurol.

[2] Traube SJ,, Wijdicks EF. Initial diagnosis and management of coma.. Emerg Med Clin N Am.

[3] Huff JS,, Stevens RD,, Weingart SD,, Smith WS. (2012). Emergency neurological life support: Approach to the patient with coma.. Neurocrit Care..

[4] Ludwig L,, McWhirter L,, Williams S,, Derry C,, Stone J. (2016;). Functional coma Handb Clin Neurol..

[5] Teasdale G,, Jennett B. (1974;). Assessment of coma and impaired consciousness. A practical scale.. Lancet.

[6] Kelly CA,, Upex A,, Bateman DN. (2004). Simple bedside assessment of level of consciousness: comparison of two simple assessment scales with the Glasgow Coma scale.. Ann Emerg Med..

[7] Wijdicks EF,, Bamlet WR,, Maramattom BV. (2005;). Validation of a new coma scale: the FOUR score.. Ann Neurol.

[8] Wijdicks EF,, Kramer AA,, Rohs T (2015;). Comparison of the full outline of unresponsiveness score and the Glasgow Coma Scale in predicting mortality in critically ill patients.. Crit Care Med.

[9] Campe GV,, Regli F,, Bogousslavsky J. (2003;). Heralding manifestations of basilar artery occlusion with lethal or severe stroke.. J Neurol Neurosurg Psychiatry.

[10] An H,, Godwin J. (2016). Flumazenil in benzodiazepine overdose.. CMAJ..

[11] Ozcan MS,, Weinberg G. (2014;). Intravenous lipid emulsion for the treatment of drug toxicity [review].. J Intensive Care Med.

